# Impairment of Bilirubin Clearance and Intestinal Interleukin-6 Expression in Bile Duct-Ligated Vitamin D Receptor Null Mice

**DOI:** 10.1371/journal.pone.0051664

**Published:** 2012-12-11

**Authors:** Michiyasu Ishizawa, Michitaka Ogura, Shigeaki Kato, Makoto Makishima

**Affiliations:** 1 Division of Biochemistry, Department of Biomedical Sciences, Nihon University School of Medicine, Tokyo, Japan; 2 Institute of Molecular and Cellular Biosciences, The University of Tokyo, Tokyo, Japan; University of Bari & Consorzio Mario Negri Sud, Italy

## Abstract

The vitamin D receptor (VDR) mediates the physiological and pharmacological actions of 1α,25-dihydroxyvitamin D_3_ in bone and calcium metabolism, cellular growth and differentiation, and immunity. VDR also responds to secondary bile acids and belongs to the NR1I subfamily of the nuclear receptor superfamily, which regulates expression of xenobiotic metabolism genes. When compared to knockout mouse investigations of the other NR1I nuclear receptors, pregnane X receptor and constitutive androstane receptor, an understanding of the role of VDR in xenobiotic metabolism remains limited. We examined the effect of VDR deletion in a mouse model of cholestasis. We performed bile duct ligation (BDL) on VDR-null mice and compared blood biochemistry, mRNA expression of genes involved in bile acid and bilirubin metabolism, cytokine production, and expression of inflammatory regulators with those of wild-type mice. VDR-null mice had elevated plasma conjugated bilirubin levels three days after BDL compared with wild-type mice. Urine bilirubin levels and renal mRNA and/or protein expression of multidrug resistance-associated proteins 2 and 4 were decreased in VDR-null mice, suggesting impaired excretion of conjugated bilirubin into urine. While VDR-null kidney showed mRNA expression of interleukin-6 (IL-6) after BDL and VDR-null macrophages had higher IL-6 protein levels after lipopolysaccharide stimulation, the induction of intestinal *Il6* mRNA expression and plasma IL-6 protein levels after BDL was impaired in VDR-null mice. Immunoblotting analysis showed that expression of an immune regulator, IκBα, was elevated in the jejunum of VDR-null mice, a possible mechanism for the attenuated induction of *Il6* expression in the intestine after BDL. Increased expression of IκBα may be a consequence of compensatory mechanisms for VDR deletion. These results reveal a role of VDR in bilirubin clearance during cholestasis. VDR is also suggested to contribute to tissue-selective immune regulation.

## Introduction

The vitamin D receptor (VDR; NR1I1) is a nuclear receptor that mediates the physiological function of the active form of vitamin D, 1α,25-dihydroxyvitamin D_3_ [1,25(OH)_2_D_3_], in numerous processes including bone and calcium metabolism, cellular growth and differentiation, immunity, and cardiovascular function [1], [2]. Upon ligand binding, VDR undergoes conformational changes that result in dynamic interaction with the heterodimer partner retinoid X receptor (RXR; NR2B) and exchange of cofactor complexes [3]. The VDR-RXR heterodimer binds preferentially to a vitamin D response element that consists of a two hexanucleotide (AGGTCA or a related sequence) direct repeat motif separated by three nucleotides, which has been identified in the regulatory regions of many target genes, including cytochrome P450 (CYP) 24A1 (gene symbol, *CYP24A1*) [4]. An everted repeat of the hexanucleotide motif separated by six, seven, eight or nine nucleotides has also been identified as vitamin D response elements in genes such as *CYP3A4* [2], [5]. VDR also acts as a receptor for secondary bile acids, such as lithocholic acid and 3-ketocholanic acid, and induces their catabolism via induction of CYP3A enzymes [6], [7]. While CYP24A1 is involved in the catabolism of 25-hydroxyvitamin D_3_ and 1,25(OH)_2_D_3_ [8], CYP3A4 catalyzes the metabolic conversion of a wide variety of xenobiotics and endogenous substrates, including 1,25(OH)_2_D_3_ [9], [10]. VDR activation has been shown to induce the expression of other enzymes and transporters involved in xenobiotic metabolism, such as multidrug resistance-associated protein 2 (MRP2; gene symbol, *Abcc2*), MRP3 (*Abcc3*), MRP4 (*Abcc4*) and sulfotransferase 2A1 [11]–[16]. VDR belongs to the NR1I nuclear receptor subfamily along with pregnane X receptor (PXR; NR1I2) and constitutive androstane receptor (CAR; NR1I3), both of which play a role in the regulation of xenobiotic metabolism [17]. Compared with PXR and CAR, however, our understanding of the role of VDR in xenobiotic metabolism remains limited.

**Figure 1:**
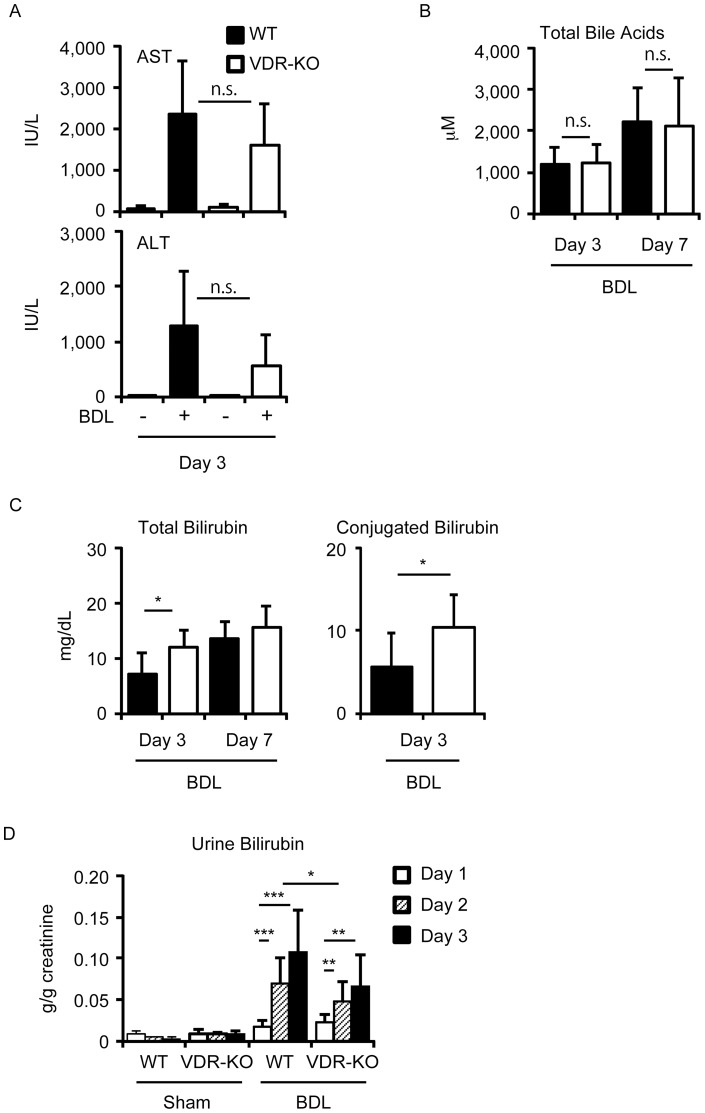
Effect of VDR deletion on plasma transaminases, total bile acids and bilirubin levels. (A) AST and ALT. (B) Total bile acids. (C) Total and conjugated bilirubin levels. Wild-type mice (WT) and VDR-null mice (VDR-KO) (n = 4–8 per group) were subjected to sham operation or BDL and plasma samples were collected 3 days and 7 days after surgery. (D) Urine bilirubin levels. Twenty-four-hour urine samples were collected 1 day, 2 days and 3 days after surgery and subjected to total bilirubin measurement. The values represent means ± S.D. *p<0.05; **p<0.01; ***p<0.001. n.s., not significant.

Cholestasis is associated with hepatic and systemic accumulation of biliary compounds, such as bile acids and bilirubin, and subsequent tissue damage [18]. Decreased secretion of bile acids into the intestine induces proliferation and translocation of intestinal bacteria, leading to endotoxemia and sepsis [19]. Endotoxin and proinflammatory cytokines can cause endotoxin-induced cholestasis and exacerbate liver injury. The bile acid-sensing nuclear receptors farnesoid X receptor (FXR; NR1H4) and PXR have been investigated in the rodent bile-duct ligation (BDL) model of cholestasis. FXR activation by synthetic ligands protects against cholestatic liver damage by decreasing expression of bile acid synthetic genes, such as *Cyp8b1*, and increasing expression of genes involved in bile acid transport, such as the bile salt export pump (BSEP; gene symbol, *Abcb11*) [20]. FXR protects hepatocytes from the toxicity of bile acid accumulation [21]. However, FXR-null mice exhibit resistance to obstructive cholestasis [22]. FXR deletion can also protect hepatocytes by facilitating the export of bile acids into blood and their renal excretion via compensatory mechanisms such as PXR activation. PXR agonists enhance bile acid detoxification by inducing the import transporter organic anion-transporting polypeptide 1A4 (OATP1A4; gene symbol, *Slco1a4*), the detoxifying enzyme CYP3A11, and the basolateral export transporter MRP3, resulting in decreased blood bile acid levels and increased urinary bile acid excretion [23]. CAR and PXR bind to response elements with overlapping specificity, and coordinately regulate xenobiotic metabolism [9]. Although there is no evidence to date that endogenous bile acids are ligands for CAR, comparison of CAR-null, PXR-null and PXR/CAR-double knockout mice shows that CAR plays a role in the elimination of toxic bile acids through a xenobiotic metabolic pathway [24]. CAR is involved in bilirubin clearance [25] and CAR activation reduces serum bilirubin levels in BDL mice [23]. VDR ligands enhance the metabolism of bile acids, particularly urinary excretion, by increasing the expression of bile acid transporter genes such as MRP4 in the mouse kidney [15]. Pharmacological VDR activation does not alter bile acid accumulation in BDL mice, but represses proinflammatory cytokine expression [14]. In this study, we investigated the *in vivo* role of VDR in the setting of cholestasis using VDR-null mice.

**Figure 2:**
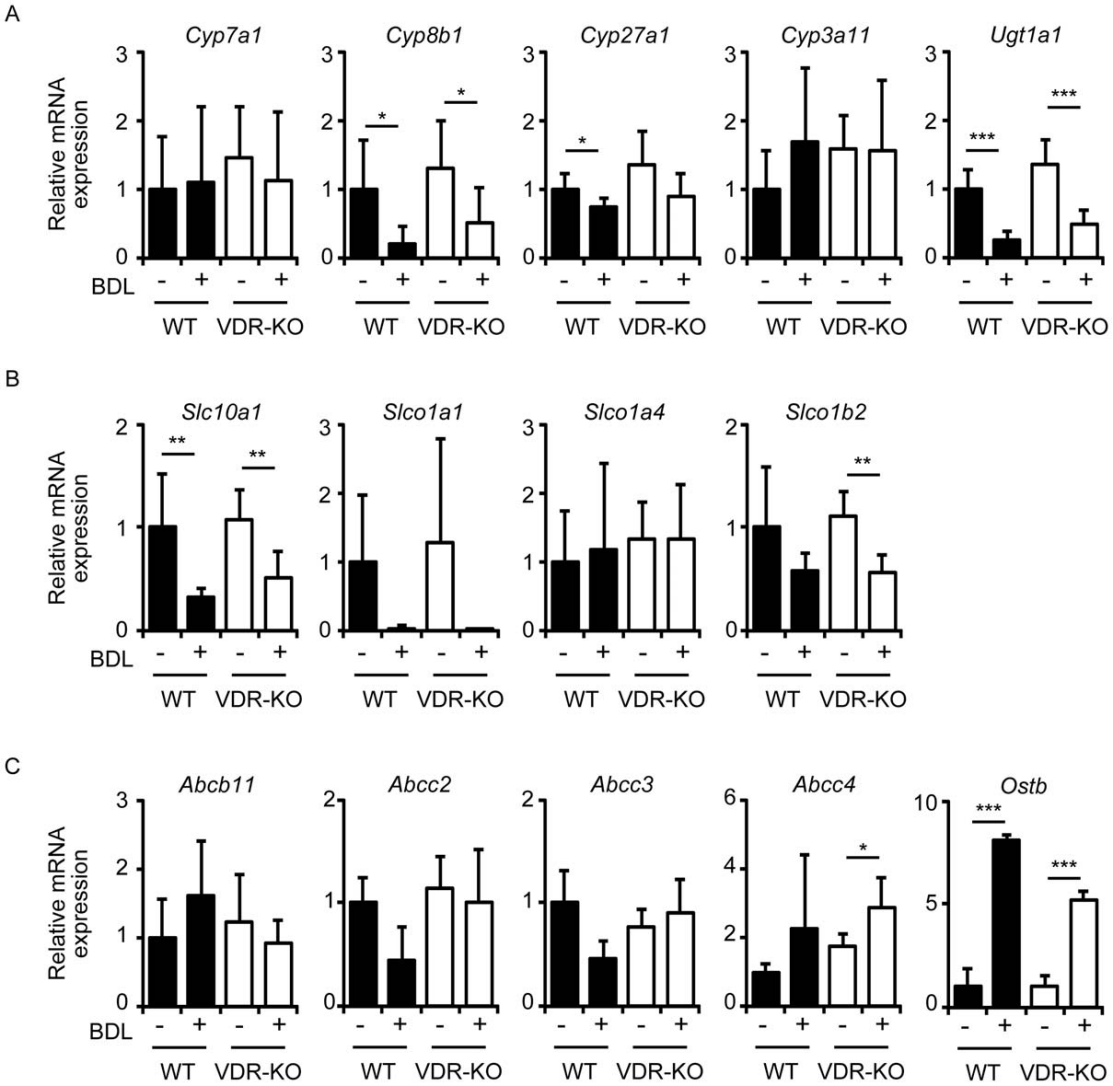
Hepatic mRNA expression of genes involved in bile acid and bilirubin metabolism. mRNA expression of enzymes (A), uptake transporters (B) and efflux transporters (C) were examined. Total RNAs were prepared from the liver of wild-type mice (WT) and VDR-null mice (VDR-KO) 7 days after sham operation or BDL. The values represent means ± S.D. *p<0.05; **p<0.01; ***p<0.001.

## Materials and Methods

### Ethics statement

The experimental protocol adhered to the Guidelines for Animal Experiments of the Nihon University School of Medicine and was approved by the Ethics Review Committee for Animal Experimentation of the Nihon University School of Medicine.

**Figure 3:**
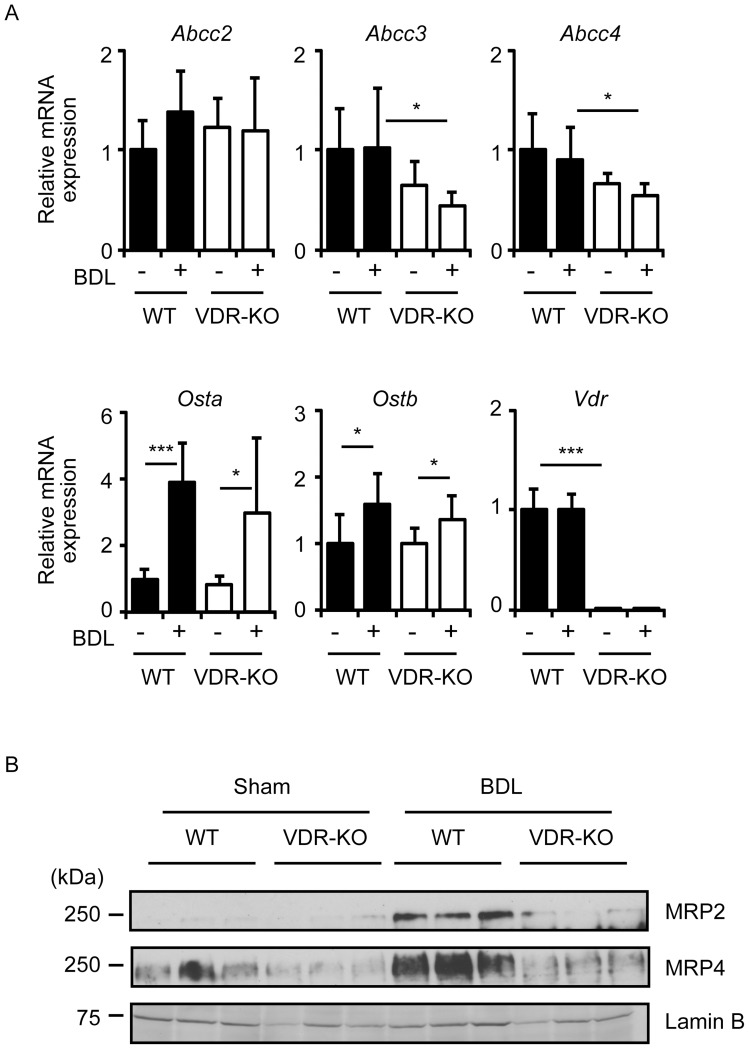
Renal mRNA and protein expression of bile acid and bilirubin transporters. (A) mRNA expression of transporters. Total RNAs were prepared from the liver of wild-type mice (WT) and VDR-null mice (VDR-KO) 7 days after sham operation or BDL. *Vdr* mRNA expression was not observed in the kidney of VDR-null mice. The values represent means ± S.D. *p<0.05; ***p<0.001. (B) Protein expression of MRP2 and MRP4. Renal proteins from the kidney of mice 3 days after surgery were subjected to immunoblotting for MRP2, MRP4 and lamin B. Each lane was loaded with 20 μg and 40 μg of membrane proteins for MRP2/4 and lamin B, respectively.

### Animal studies

VDR-null (*Vdr* ^−/−^) mice and control wild-type (*Vdr* ^+/+^) mice were obtained by breeding C57BL/6J *Vdr* ^+/−^ mice and were fed CLEA Rodent Diet CE-2 (CLEA Japan, Tokyo, Japan) supplemented with 2% calcium, 1.5% phosphate, and 20% lactose [26], [27]. All mice were maintained under controlled temperature (23±1 C) and humidity (45–65%) with free access to water and food. Experiments were conducted with female mice between 7 and 10 weeks of age. BDL and sham surgery were performed as reported previously [14]. Plasma, liver, kidney, intestine and spleen samples were collected 3 and 7 days after surgery. The 24-hour urine was collected by using mouse metabolic cages. Total and conjugated bilirubin, aspartate aminotransferase (AST), alanine aminotransferase (ALT) and total bile acids were quantified with Bilirubin BII-Testwako, Transaminase CII-Testwako and Total bile acid-Testwako (Wako Pure Chemicals, Osaka, Japan), respectively. Urinary bilirubin concentrations were normalized with creatinine levels. Cytokine levels were determined with enzyme-linked immunosorbent assay kits (R&D Systems Inc., Minneapolis, MN). The optical density was measured with a Flex Station III microplate reader (Molecular Devices, Sunnyvale, CA).

**Figure 4:**
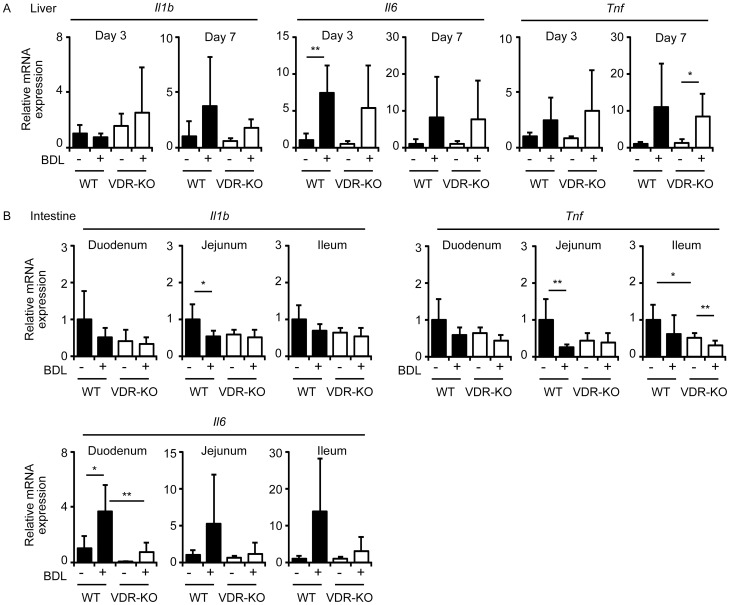
mRNA expression of inflammatory cytokines in the liver (A) and intestine (B). Total RNAs were prepared from the liver of wild-type mice (WT) and VDR-null mice (VDR-KO) 3 days and 7 days after sham operation or BDL. The values represent means ± S.D. *p<0.05; **p<0.01.

### Peritoneal macrophages

Peritoneal macrophages were obtained from mice treated with 3% thioglycollate intraperitoneally for 4 days [28]. Cells were cultured in RPMI 1640 medium containing 10% fetal bovine serum, 100 units/ml penicillin and 100 mg/ml streptomycin for 24 hours and then treated with or without 10 ng/ml lipopolysaccharide (LPS) for 12 hours.

**Figure 5:**
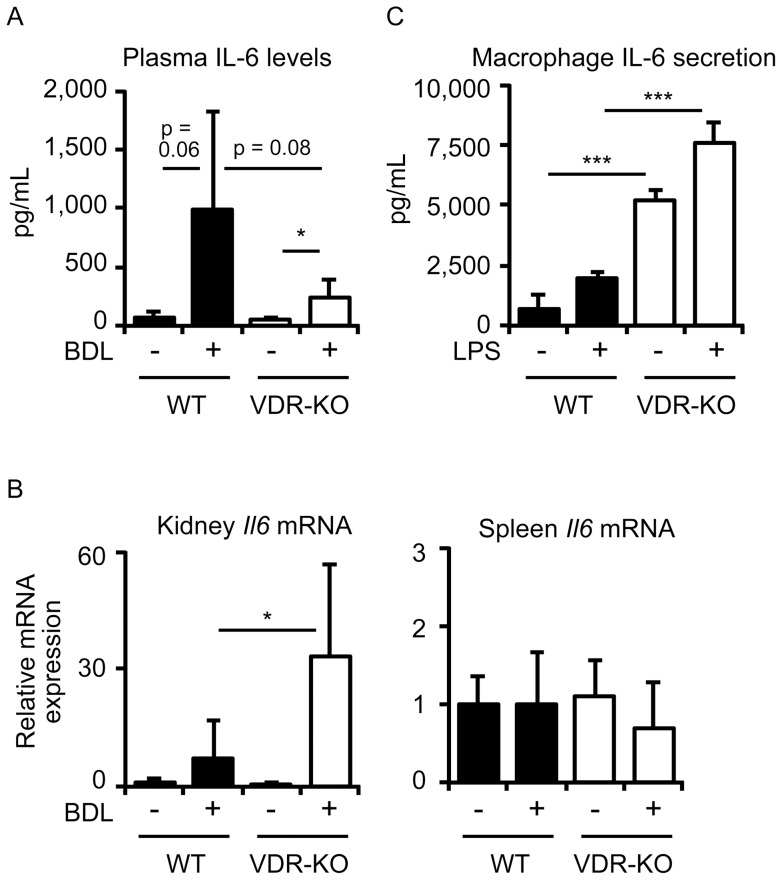
Plasma IL-6 levels, renal and splenic *Il6* mRNA expression and IL-6 production in peritoneal macrophages. Plasma IL-6 levels (A) and *Il6* mRNA expression in the kidney and spleen (B) of mice with BDL. Plasma and total RNAs from the kidney and spleen were prepared from wild-type mice (WT) and VDR-null mice (VDR-KO) 3 days after sham operation or BDL. (C) Secreted IL-6 production in peritoneal macrophages. Peritoneal macrophages were treated with 10 ng/ml LPS for 12 hours and secreted IL-6 protein levels in media were measured. The values represent means ± S.D. *p<0.05; ***p<0.001.

### Reverse transcription and real-time quantitative polymerase chain reaction

Total RNAs from samples were prepared by the acid guanidine thiocyanate-phenol/chloroform method [14]. cDNAs were synthesized using the ImProm-II Reverse Transcription system (Promega Corporation, Madison, WI). Intron-spanning primers were as follows: *Abcc3* (GenBank accession no. NM_029600), 5′- GCC AAC TTC CTC CGA AAC TA-3′ and 5′- CTT GCG GAC CTC GTA GAT GG-3′; *Ugt1a1* (GenBank accession no. NM_201645), 5′- TCT GAG CCC TGC ATC TAT CT-3′ and 5′- AGA GGC GTT GAC ATA GGC TT-3′; *Socs1* (GenBank accession no. NM_009896), 5′-GTG GTT GTG GAG GGT GAG AT-3′ and 5′-CCC AGA CAC AAG CTG CTA CA-3′; *Socs3* (GenBank accession no. NM_007707), 5′-TTG TCG GAA GAC TGT CAA CG-3′ and 5′-GAG CAT CAT ACT GAT CCA GG-3′. Other primer sequences have reported previously [14], [27], [29]. The mRNA values were normalized to the amount of glyceraldehyde-3-phosphate dehydrogenase mRNA.

**Figure 6:**
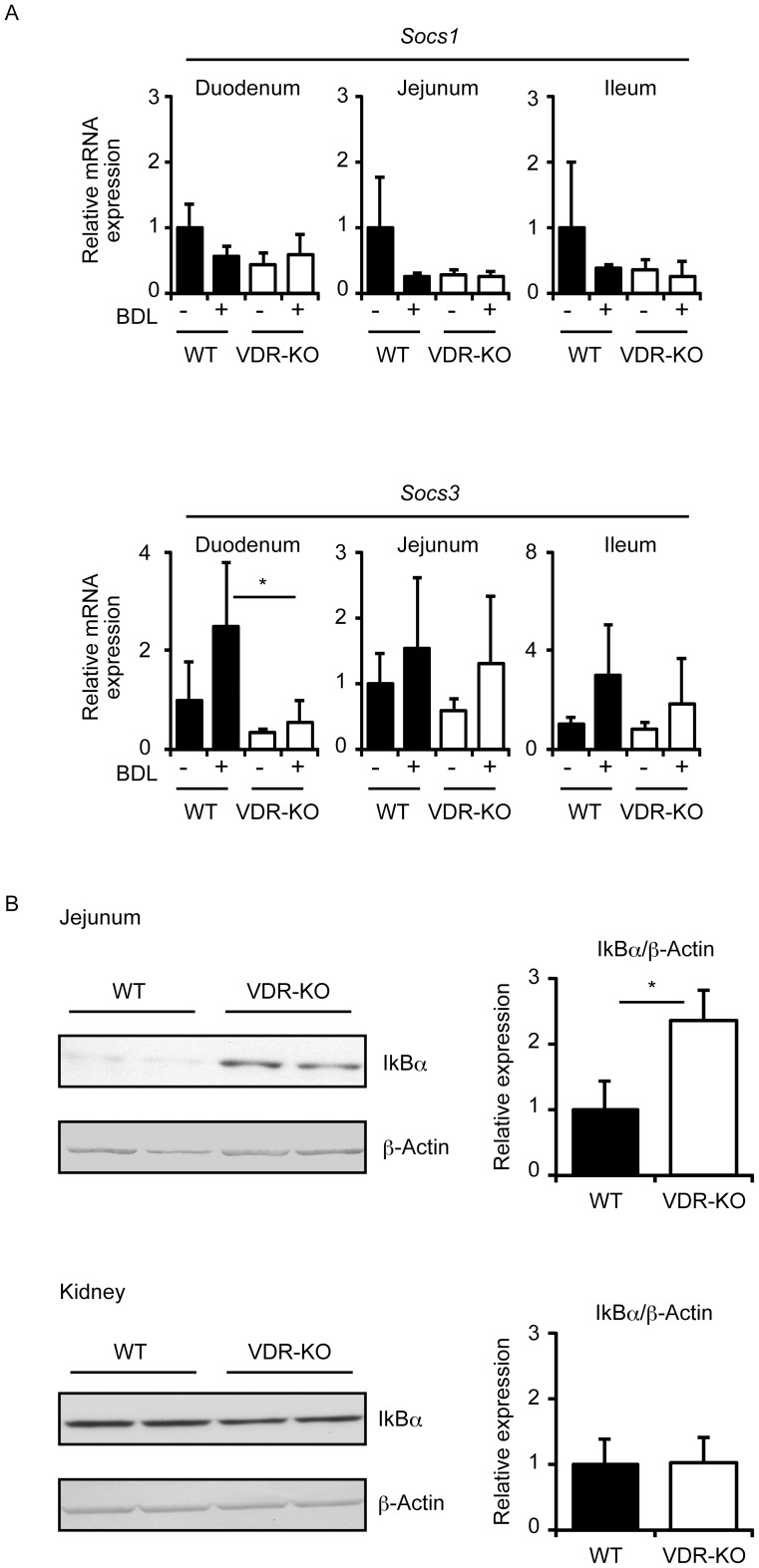
VDR deletion increases intestinal IκBα expression but not *Socs1* or *Socs3* mRNA expression. (A) *Socs1* and *Socs3* mRNA expression in the intestine. Total RNAs were prepared from the liver of wild-type mice (WT) and VDR-null mice (VDR-KO) 3 days after sham operation or BDL. (B) IκBα protein levels in the jejunum and kidney. Each lane was loaded with 50 μg (jejunum) or 100 μg (kidney) of whole tissue lysates. Band intensity was quantified with Image J 1.45 (n = 3–4). The values represent means ± S.D. *p<0.05.

### Immunoblotting

For MRP4 expression, membrane fractions from the kidney were prepared as reported previously [30]. For IκBα expression, tissue samples form the jejunum and kidney were homogenized in buffer (50 mM Tris, pH 8.0, 150 mM NaCl, 0.5% deoxycholate, 0.1% sodium dodecyl sulfate, 1% NP40) containing a protease inhibitor cocktail, and centrifuged to remove debris. Western blot analysis was performed using anti-MRP2 antibody (Santa Cruz Biotechnology, Santa Cruz, CA), an anti-MRP4 antibody (Santa Cruz Biotechnology), an anti-lamin B antibody (Santa Cruz Biotechnology), an anti-IκBα antibody (Santa Cruz Biotechnology) and an anti-β-actin antibody (Sigma-Aldrich, St. Louis, MO), visualized with an alkaline phosphatase conjugate substrate system or an enhanced chemiluminescence detection system [15], [31], [32].

### Statistical analysis

All values are shown as mean ± S.D. The unpaired two-group Student's t test was performed to assess significant differences.

## Results

### VDR deletion increases plasma conjugated bilirubin levels in BDL mice

To examine the effect of VDR deletion on cholestasis, we performed BDL and sham surgery on wild-type mice and VDR-null mice. Plasma AST and ALT levels increased drastically 3 days after BDL, while sham surgery had no effect (Fig. 1A). There was no significant difference in AST and ALT levels between wild-type mice and VDR-null mice (Fig. 1A). Increased total bile acid levels at both day 3 and day 7 were not changed by VDR deletion (Fig. 1B). Interestingly, plasma total bilirubin levels 3 days after BDL were significantly elevated in VDR-null mice when compared to wild-type mice (Fig. 1C). BDL increased urine bilirubin levels in both wild-type and VDR-null mice, while sham surgery had no effect (Fig. 1D). VDR-null mice had lower concentrations of urine bilirubin than wild-type mice at day 2 after BDL, and a similar pattern was observed at day 3 although it did not reach statistical significance (Fig. 1D). There was no significant difference in plasma total bilirubin levels 7 days after BDL between wild-type mice and VDR-null mice (Fig. 1C), a finding that may be due to saturation of plasma bilirubin levels. Accumulation of conjugated bilirubin contributed to increased plasma bilirubin levels in VDR-null mice treated with BDL (Fig. 1C). Thus, VDR deletion decreases the urinary clearance of conjugated bilirubin from blood in BDL mice.

We next examined the mRNA expression of genes involved in metabolism and transport of bile acids and bilirubin in the liver of mice 7 days after sham or BDL surgery. *Cyp7a1*, *Cyp8b1* and *Cyp27a1* encode bile acid synthetic enzymes [33]. Although previous reports have shown that BDL increases *Cyp7a1* expression [29], [34], [35], there was no difference in *Cyp7a1* expression among sham-operated wild-type, BDL wild-type, sham-operated VDR-null and BDL VDR-null mice (Fig. 2A). As reported previously [14], [24], BDL decreased *Cyp8b1* expression in wild-type mice. The decrease in *Cyp8b1* mRNA levels in VDR-null mice subjected to BDL was similar to that in wild-type mice (Fig. 2A). *Cyp27a1* expression was slightly decreased by BDL in wild-type mice and VDR deletion did not affect *Cyp27a1* expression (Fig. 2A). *Cyp3a11* is a target gene of VDR, PXR and CAR [6], [36], [37], and BDL has been shown to increase *Cyp3a11* expression [29], [38], [39]. In our experiments, BDL tended to increase *Cyp3a11* expression but this effect was not significant due to large variation (Fig. 2A). BDL did not change *Cyp3a11* mRNA levels in VDR-null mice. A CAR target gene, *Ugt1a1*, encodes a bilirubin-conjugating enzyme [25]. *Ugt1a1* expression was decreased to a similar extent in the liver 7 days after BDL in both wild-type and VDR-null mice (Fig. 2A).

Na^+^/taurocholate cotransporting polypeptide (NTCP; encoded by the *Slc10a1* gene) is involved in bile acid uptake at the basolateral membrane of hepatocytes [18]. OATP1A1 (encoded by the *Slco1a1* gene), OATP1A4 (encoded by the *Slco1a4* gene) and OATP1B2 (encoded by the *Slco1b2* gene) are basolateral transporters for the uptake of bile acids and bilirubin in the liver [18], [40]. As reported previously [14], [24], [41], BDL decreased *Slc10a1* expression in wild-type mice (Fig. 2B). BDL also tended to decrease *Slco1a1* and *Slco1b2* expression but these differences were not significant, while a significant decrease in *Slco1b2* expression was observed in BDL VDR-null mice compared with sham-operated VDR-null mice (Fig. 2B). *Slco1a4* mRNA levels were not changed after BDL. VDR deletion did not affect expression of these uptake transporters in the liver of sham-operated and BDL mice (Fig. 2B).

BSEP (encoded by the *Abcb11* gene) and MRP2 (encoded by the *Abcc2* gene) are localized in the canalicular membrane of hepatocytes and excrete bile acids into bile, while MRP3 (encoded by the *Abcc3* gene), MRP4 (encoded by the *Abcc4* gene), organic solute transporter (OST) α(encoded by the *Osta* gene), and OSTβ (encoded by the *Ostb2* gene) are found at the hepatocyte basolateral membrane and play a role in the alternative excretion of bile acids into the systemic circulation [18]. There was no difference in *Abcb11* expression in sham-operated wild-type, BDL wild-type, sham-operated VDR-null and BDL VDR-null mice (Fig. 2C). BDL tended to decrease *Abcc2* and *Abcc3* expression in wild-type mice but not in VDR-null mice (Fig. 2C). BDL increased *Abcc4* expression in VDR-null mice but this effect was not significant in wild-type mice (Fig. 2C). BDL increased *Ostb* expression in both wild-type and VDR-null mice (Fig. 2C). There was no significant difference in the expression of these genes in wild-type and VDR-null mice.

### VDR deletion decreases MRP2 and MRP4 expression in the kidney of BDL mice

Hepatic MRP2 and MRP3 have been reported to be involved in the transport of conjugated bilirubin in mice [42]–[45]. In the kidney, MRP2 and MRP4 are expressed on the apical membrane in proximal tubule cells, while MRP3 localizes to the basolateral membrane of distal convoluted tubules [46]. We first examined mRNA expression of renal MRP transporters. There was no difference in *Abcc2* expression in sham-operated wild-type, BDL wild-type, sham-operated VDR-null and BDL VDR-null mice (Fig. 3A). Expression of *Abcc3* and *Abcc4* tended to decrease in VDR-null mice compared with wild-type mice following sham operation. Expression levels of these transporters were significantly lower in VDR-null mice with BDL than in sham-operated, wild-type mice (Fig. 3A). The heterodimeric bile acid transporter genes, *Osta* and *Ostb*, were increased by BDL to similar levels in wild-type and VDR-null mice (Fig. 3A). We confirmed the loss of *Vdr* mRNA expression in the kidney of VDR-null mice (Fig. 3A). Next, we examined protein expression of the renal apical efflux transporters MRP2 and MRP4. MRP2 and MRP4 protein levels were elevated by BDL in wild-type mice (Fig. 3B). Interestingly, protein expression of MRP2 and MRP4 was not changed after BDL in VDR-null mice (Fig. 3B). Thus, VDR deletion impairs the renal expression of MRP2, MRP3 and MRP4 at the mRNA and/or protein levels.

### VDR deletion changes intestinal cytokine mRNA expression after BDL

Since we have previously reported that pharmacological VDR activation represses inflammatory cytokine expression in BDL mice [14], we examined mRNA expression of inflammatory cytokines in the liver and intestine. In contrast to pharmacological VDR activation, VDR deletion did not affect BDL-induced changes in hepatic expression of interleukin-1b (gene symbol, *Il1b*), interleukin-6 (IL-6: gene symbol, *Il6*) and tumor necrosis factor (gene symbol, *Tnf*) (Fig. 4A), suggesting that VDR activity regulated by endogenous ligands is not involved in hepatic cytokine mRNA expression after BDL. Interestingly, BDL decreased jejunal *Il1b* expression in wild-type mice but not in VDR-null mice (Fig. 4B). Duodenal and ileal *Il1b* expression tended to decrease after BDL in wild-type mice and did not change in VDR-null mice. Similar to *Il1b*, jejunal *Tnf* expression decreased after BDL in wild-type mice but not in VDR-null mice (Fig. 4B). Duodenal *Tnf* tended to decrease after BDL in both wild-type mice and VDR-null mice. Ileal *Tnf* decreased after BDL in VDR-null mice but not to a significant degree in wild-type mice. In contrast to *Il1b* and *Tnf*, duodenal, jejunal and ileal *Il6* expression increased after BDL in wild-type mice, although this was not significant due to large variation between animals (Fig. 4B). Intestinal *Il6* expression was not induced by BDL in VDR-null mice (Fig. 4B).

### VDR deletion modifies *Il6* mRNA expression in a tissue-selective manner

VDR activation suppresses hepatic *Il6* expression and plasma IL-6 levels increased by BDL [14], and bacterial infection increases serum IL-6 levels to a greater extent in VDR-null mice [47]. From these findings, we have hypothesized that BDL induces higher *Il6* expression in VDR-null mice than wild-type mice. However, we observed less *Il6* induction in the intestine of VDR-null mice after BDL (Fig. 4B). We compared plasma IL-6 protein levels after BDL in wild-type and VDR-null mice. BDL increased plasma IL-6 levels from 63±58 to 986±845 pg/ml in wild-type mice and from 61±15 to 242±157 pg/ml in VDR-null mice (Fig. 5A), results consistent with the intestinal *Il6* mRNA expression data (Fig. 4B). In contrast, kidney *Il6* mRNA levels were higher in VDR-null mice than wild-type mice (Fig. 5B). There was no difference in splenic *Il6* expression among sham-operated wild-type, BDL wild-type, sham-operated VDR-null and BDL VDR-null mice (Fig. 5B). We isolated peritoneal macrophages from wild-type and VDR-null mice and examined IL-6 production after LPS stimulation. VDR-null macrophages produced higher IL-6 protein levels than wild-type macrophages even in the absence of LPS stimulation (Fig. 5C). LPS treatment induced more IL-6 protein secretion from VDR-null macrophages than wild-type macrophages. Thus, VDR deletion results in impaired IL-6 production in a tissue-selective manner.

### VDR deletion is associated with increased IκBα protein levels in the intestine

VDR null mice have increased serum IL-6 levels and VDR deletion elevates nuclear factor (NF)-κB activity in intestinal epithelia and mouse embryonic fibroblasts (MEFs) [47]. In agreement with these findings, VDR-null peritoneal macrophages showed elevated IL-6 secretion (Fig. 5C). However, intestinal *Il6* mRNA expression and plasma IL-6 levels were less increased after BDL in VDR-null mice compared with wild-type mice (Fig. 4B and Fig. 5A). We speculated that while VDR primarily regulates inflammatory responses by suppressing NF-κB activity, VDR deletion may induce compensatory immunoregulatory mechanisms in the intestine. Since suppressor of cytokine signaling (SOCS) 1 and SOCS3 have been reported to inhibit LPS-induced IL-6 production [48], [49], we examined the expression of *Socs1* and *Socs3* in the intestine. There was no difference in *Socs1* expression in sham-operated wild-type, BDL wild-type, sham-operated VDR-null and BDL VDR-null mice, although BDL tended to decrease *Socs1* expression in wild-type mice (Fig. 6A). BDL tended to increase duodenal, jejunal and ileal *Socs3* expression. Interestingly, duodenal *Socs3* mRNA levels in VDR-null mice were lower than in wild-type mice (Fig. 6A), a pattern similar to that observed for *Il6* expression (Fig. 4B). Jejunal and ileal *Socs3* expression tended to increase after BDL in VDR-null mice. Decreased *Il6* induction in the intestine of VDR-null mice with BDL cannot be explained by the expression of *Socs1* or *Socs3*. Intestinal *Socs3* expression may be regulated by of cytokines such as IL-6 [50].

NF-κB induces mouse *Il6* transcription [51], and IκBα binds to NF-κB and represses its transactivation activity [52]. We examined IκBα protein levels in jejunum and kidney by immunoblotting. Interestingly, jejunal IκBα protein levels were higher in VDR-null mice than wild-type mice, while its expression was similar in the kidney of VDR-null mice and wild-type mice (Fig. 6B). Thus, higher IκBα protein levels are associated with reduced *Il6* induction in the jejunum of VDR-null mice.

## Discussion

In this study, we examined the effects of VDR deletion in a mouse model of cholestasis and found that VDR-null mice show increased plasma bilirubin levels and reduced intestinal *Il6* induction after BDL compared to wild-type mice. Under physiological conditions, conjugated bile acids and conjugated bilirubin are secreted from the canalicular membrane of hepatocytes into bile. Under cholestatic conditions, however, these compounds are effluxed from the basolateral membrane of hepatocytes to the blood circulation and are then excreted into the urine via renal transporters, a major alternative elimination route [18]. VDR-null mice accumulated higher levels of conjugated bilirubin in the plasma than wild-type mice (Fig. 1). We examined the effect of 1α-hydroxyvitamin D_3_ treatment on hyperbilirubinemia induced by intravenous bilirubin infusion in wild-type mice and found that VDR activation did not increase the rate of clearance of exogenous bilirubin (unpublished data). Injected bilirubin is glucuronidated in hepatocytes and excreted into bile. Expression of *Ugt1a1* was similar between wild-type mice and VDR-null mice (Fig. 2). Thus, VDR deletion disrupts the clearance of conjugated bilirubin from blood rather than bilirubin conjugation in BDL mice.

MRP2, MRP3 and MRP4 are involved in transport of bile acids and/or bilirubin in the liver and kidney [18], [46]. MRP2 is localized at the canalicular membrane of hepatocytes and MRP2-null mice show increased serum conjugated bilirubin [42], [43], indicating that MRP2 plays a role in biliary excretion of conjugated bilirubin. MRP3 and MRP4 are expressed at the basolateral hepatocyte membrane [46]. BDL results in reduced accumulation of serum conjugated bilirubin in MRP3-null mice [44], [45]. MRP4-null mice have reduced serum bile acids but similar conjugated bilirubin levels after BDL [53]. MRP3, which is upregulated in MRP4-null mice, is insufficient for basolateral bile acid secretion [53], but may adequately compensate for bilirubin excretion. As reported previously [14], [24], [29], [35], [54], BDL did not increase hepatic *Abcc2* mRNA expression in wild-type mice (Fig. 2). Hepatic *Abcc3* and *Abcc4* mRNA have been reported to be upregulated after BDL [29], [35], [54]. In our results, BDL tended to increase *Abcc4* but not *Abcc3* expression in the liver of wild-type mice (Fig. 2). Potential confounding factors of BDL, such as secondary inflammation, may influence *Abcc3* expression [55]. VDR deletion had a modest or no effect on the regulation of hepatic genes involved in the metabolism of bile acids and bilirubin during cholestasis (Fig. 2). VDR ligand treatment does not induce hepatocyte target gene expression due to the low expression of VDR in liver [56], [57]. While VDR activation does not alter bile acid accumulation in BDL mice [14], it enhances urinary excretion of bile acids by increasing the expression of bile acid transporter genes in mice fed chow supplemented with chenodeoxycholic acid [15]. These findings suggest that an increase in plasma conjugated bilirubin in VDR-null mice with BDL is due to impaired expression of renal transporters of bilirubin.

In the kidney, MRP2 and MRP4 are localized to the apical tubular membrane, while MRP3 is expressed in the basolateral membrane of the distal convoluted tubule [18], [46]. We observed reduced MRP2 protein levels and MRP4 mRNA and protein levels in the kidney of VDR-null mice (Fig. 3), consistent with our previous reports that show that pharmacological VDR activation increases mRNA and/or protein expression of MRP2 and MRP4 [14], [15]. There were some discrepancies between mRNA and protein levels of MRP2 and MRP4 (Fig. 3). 1,25(OH)_2_D_3_ treatment increases MRP2 and MRP4 protein levels without changing mRNA levels in the rat intestine [16]. 1,25(OH)_2_D_3_ also induces MRP2 and MRP4 protein expression with increasing *ABCC2* mRNA levels but without affecting *ABCC4* mRNA expression in human intestinal Caco-2 cells [13]. VDR activation may induce MRP2 and MRP4 expression through both transcriptional and posttranscriptional mechanisms. MRP2 plays a role in biliary excretion of conjugated bilirubin [42], [43]. Although MRP4-null mice have serum bilirubin at the same levels as wild-type mice after BDL [53], a compensatory increase in MRP3 expression may mask the effect of MRP4 deficiency on bilirubin transport. MRP3-null mice exhibit BDL-induced liver damage at a similar extent to wild-type mice and have increased MRP4 expression [44], [45]. Thus, hepatic MRP3 and MRP4 may have overlapping roles in basolateral efflux transport. In the kidney, in contrast to the basolateral transporter MRP3, the apical transporters MRP2 and MRP4 play a role in urinary excretion of endogenous and exogenous chemicals. Therefore, reduced induction of MRP2 and possibly MRP4 in the kidney could cause impaired urinary clearance of conjugated bilirubin in VDR-null mice, although an involvement of other VDR-dependent mechanisms cannot be ruled out. Thus, VDR may play a role in xenobiotic metabolism by regulating the expression of renal transporter genes.

In contrast to previous reports showing that BDL increases *Cyp7a1* expression [29], [34], [35], BDL did not change *Cyp7a1* expression (Fig. 2). BDL blocks bile flow into the intestine and decreases expression of fibroblast growth factor 15, a target gene of the bile acid receptor FXR [34]. Since secreted fibroblast growth factor 15 inhibits *Cyp7a1* expression in the liver, BDL can increase *Cyp7a1* expression [34]. On the other hand, accumulated bile acids in cholestasis induce activation of FXR and PXR in the liver [58]. FXR induces small heterodimer partner to inhibit *Cyp7a1* expression and PXR also inhibits *Cyp7a1* transcription. Furthermore, inflammatory cytokines inhibit *Cyp7a1* expression [58]. In agreement with our results (Fig. 2), *Cyp7a1* mRNA levels have been shown to be unchanged after BDL [14], [54]. BDL has also been shown to decrease *Cyp7a1* expression [59]. Thus, confounding factors associated with experimental conditions may affect *Cyp7a1* expression.

BDL-induced *Il6* expression in the intestine was diminished in VDR-null mice (Fig. 4). Plasma IL-6 levels after BDL were also lower in VDR-null mice when compared to wild-type mice (Fig. 5). These findings were unexpected because we have reported that 1α-hydroxyvitamin D_3_ treatment suppresses the increase in plasma IL-6 seen after BDL [14]. VDR-null mice have increased IL-6 production after *Salmonella* infection and VDR deletion is associated with elevated NF-κB in intestinal epithelia [47]. In *Vdr* ^−/−^ MEFs, the basal level of IκBα protein is markedly decreased when compared to *Vdr* ^+/−^ MEFs, leading to increased NF-κB transcriptional activity [60]. NF-κB is a major inducer of *Il6* transcription [51], [61]–[63]. VDR activation suppresses NF-κB activity by increasing IκBα levels in keratinocytes and macrophages [64], [65], consistent with our previous report that shows that VDR activation decreases production of inflammatory cytokines, including IL-6 [14]. IL-6 production by peritoneal macrophages and renal *Il6* mRNA expression were augmented in VDR-null mice (Fig. 5), supporting a proinflammatory state in VDR-null cells [47]. In contrast to peritoneal macrophages and kidney, the intestine of VDR-null mice exhibited less *Il6* induction (Fig. 4). Plasma IL-6 protein levels exhibited a similar pattern to intestinal *Il6* mRNA expression (Fig. 5), although we cannot rule out other tissue sources of IL-6 production. Interestingly, IκBα protein levels were higher in the jejunum of VDR-null mice than wild-type mice (Fig. 6). *Vdr* ^−/−^ MEFs have increased levels of IκBα mRNA, and decreased levels of IκBα protein, possibly due to increased protein degradation [66]. The VDR-null mice used in our experiments may maintain intestinal homeostasis by increasing IκBα protein levels. Through this mechanism, they may be resistant to *Il6* induction after BDL when compared to wild-type mice.

VDR is activated by lithocholic acid and its derivatives [6], [67], [68]. While VDR deletion increased conjugated bilirubin, it did not affect total bile acids in the plasma (Fig. 1). Major bile acids that accumulate after BDL are cholic acid, β-muricholic acid and ω-muricholic acid [14]. These bile acids fail to activate VDR both in cells and mouse tissues [6], [14]. Although treatment of mice with 1α-hydroxyvitamin D_3_ induces expression of VDR target genes in the liver, kidney and intestine, it is not enough to reduce accumulated bile acids [14]. In the context of BDL, VDR may fail to function as a regulator of bile acid metabolism. Lithocholic acid is a secondary bile acid generated by intestinal microflora and has minimal intestinal absorption [15], [69], suggesting that intestinal VDR senses lithocholic acid signaling from bacteria. Since VDR regulates intestinal immunity [47], [70], it may mediate a host-bacterial signaling pathway through lithocholic acid or related compounds. Vitamin D signaling is also important in maintaining intestinal immunity [71]. VDR-null mice may develop an altered microflora-host immune, leading to differences in cytokine expression. Further investigation is needed to elucidate the mechanism of increased intestinal IκBα expression in VDR-null mice.
